# Children with obesity have cardiac remodeling and dysfunction: a cine DENSE magnetic resonance imaging study

**DOI:** 10.1186/1532-429X-17-S1-Q57

**Published:** 2015-02-03

**Authors:** Cassi M Binkley, Linyuan Jing, Jonathan D Suever, Nivedita Umasankar, Gregory J Wehner, Sean M Hamlet, David Powell, Aurelia Radulescu, Frederick H Epstein, Brandon K Fornwalt

**Affiliations:** 1 Physiology, University of Kentucky, Lexington, KY, USA; 2 Pediatrics, University of Kentucky, Lexington, KY, USA; 3 Biomedical Engineering, University of Kentucky, Lexington, KY, USA; 4 Radiology, University of Kentucky, Lexington, KY, USA; 5 Biomedical Engineering, University of Virginia, Charlottesville, VA, USA

## Background

Obesity affects one in five children in the US and these children tend to maintain excess weight into adulthood. It has recently been shown that childhood obesity is associated with both cardiac remodeling (hypertrophy) and contractile dysfunction. However, the etiology of these cardiac changes is not well understood. We hypothesized that cardiac remodeling and dysfunction could not be entirely explained by elevations in blood pressure and that excess abdominal and epicardial adiposity may also correlate with cardiac changes.

## Methods

Healthy weight (5^th^-85^th^ percentile for age and height) and obese (≥95^th^ percentile) children between the ages of 8 and 18 were recruited. All subjects underwent MRI on a 3T Siemens Tim Trio scanner using a 6 element chest coil and 24 element spine coil. Cine SSFP images were used to quantify cardiac remodeling (left ventricular mass and thickness) and epicardial adiposity. Displacement Encoding with Stimulated Echoes (DENSE) was used to quantify cardiac strains and torsion with base, mid, and apical short axis images and 2- and 4-chamber long axis views. Phase contrast imaging was used to measure diastolic filing velocities and T1 weighted imaging was used to quantify subcutaneous and visceral adiposity using a transverse slice at the L4-L5 disc. Blood pressure was averaged from two readings at a resting state by auscultation, using an aneroid sphygmomanometer, and an appropriate sized cuff.

## Results

**Figure 1:**
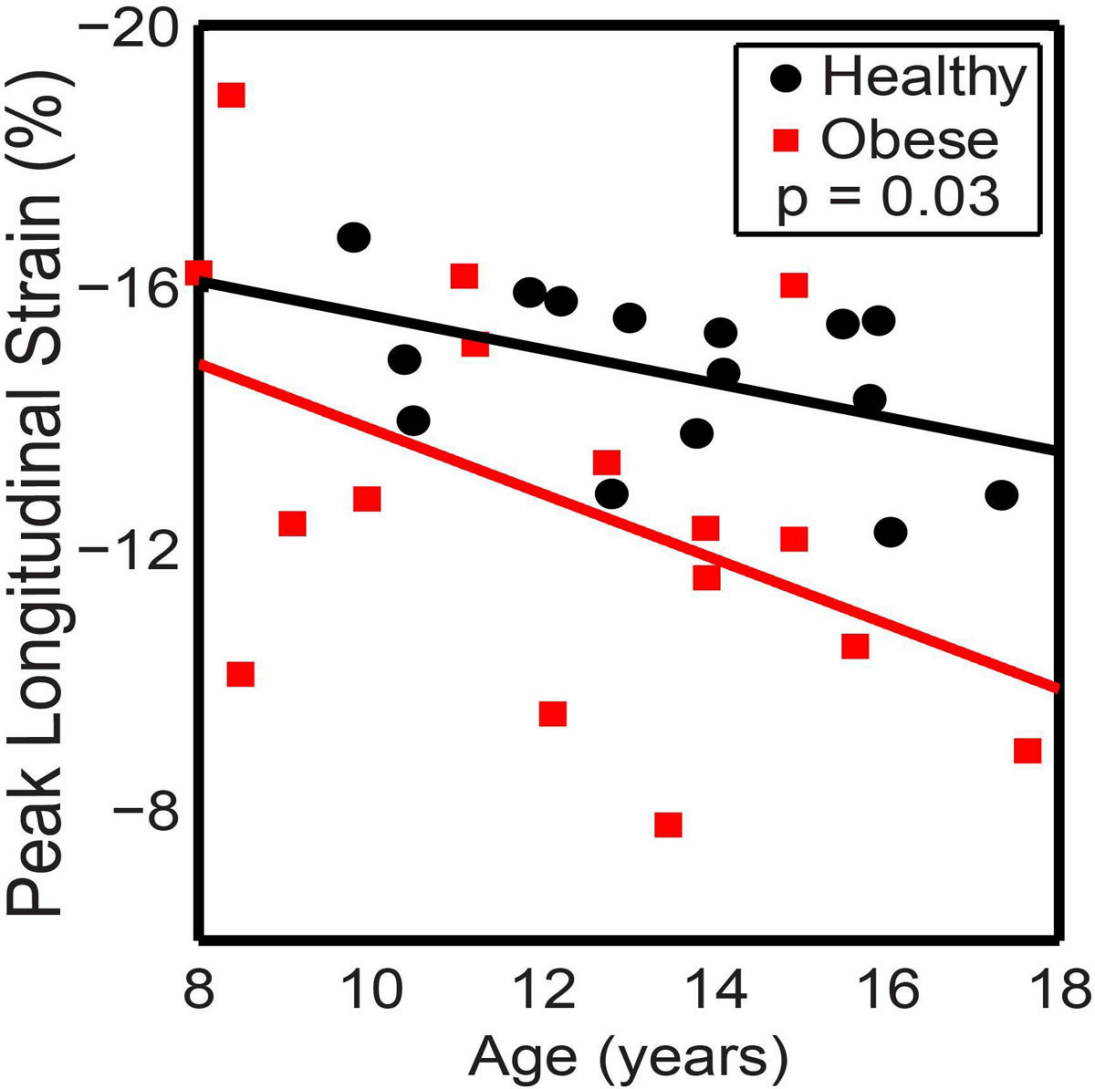
Children with obesity have reduced peak longitudinal strain compared to healthy controls.

Sixteen obese children (ages 12.2 ± 2.9, 63% female) and 15 healthy controls (ages 13.5 ± 2.3, 60% female) were enrolled. Children with obesity had a left ventricular mass index (LVMI) that was increased by 28% compared to healthy controls (p< 0.001). Children with obesity also showed a 40% reduction in E/A ratio compared to the control group (p = 0.01). There were no differences in ejection fraction, end systolic or end diastolic volumes between the two groups. However, we observed a 14% reduction in global, peak longitudinal strain in children with obesity (p = 0.03, Figure 1), suggesting impairment of contractile function in the left ventricle. Additionally, children with obesity had more than double the amount of visceral adiposity, subcutaneous adiposity, and epicardial adipose tissue. Systolic blood pressure was weakly positively correlated with LVMI (r = 0.4, p = 0.025) and longitudinal strain (r = 0.54, p = 0.002). Visceral and deep subcutaneous adiposity were also weakly positively correlated with LVMI (r = 0.37, p = 0.04 and r = 0.42, p = 0.02, respectively). Epicardial adiposity was more strongly correlated with longitudinal strain (r = 0.6, p < 0.001), suggesting that epicardial fat could contribute to reduced contractile function.

**Table 1:** Children with obesity have cardiac remodeling and dysfunction

	Obese, n = 16	Healthy, n = 15	p value
Left Ventricular Mass Index (g/m^2.7)	29 ± 4	22 ± 3	*<0.001

Average Left Ventricular Thickness (mm)	6 ± 0.8	5.3 ± 0.8	*0.013

E/A Ratio	2.2 ± 0.9	3.3 ± 1.2	*0.01

Ejection Fraction (%)	58 ± 6	54.8 ± 6	0.19

End Diastolic Volume (mL)	132 ± 30	144 ± 35	0.34

End Systolic Volume (mL)	56 ± 14	65 ± 17	0.14

Peak Radial Strain (%)	33.5 ± 9	28.6 ± 12	0.22

Peak Circumferential Strain (%)	17.8 ± 2	18.3 ± 2	0.51

Peak Longitudinal Strain (%)	12.7 ± 3	14.6 ± 1	*0.03

Torsion (°/cm)	4 ± 1	3.6 ± 2	0.26

Systolic Blood Pressure (mmHg)	115 ± 10	105 ± 8	*0.007

Diastolic Blood Pressure (mmHg)	75 ± 7	68 ± 5	*0.005

Mean Arterial Pressure (mmHg)	88 ± 7	80 ± 5	*0.002

Subcutaneous Fat (% of body cavity)	60 ± 8	30 ± 13	*<0.001

Visceral Fat (% of visceral cavity)	34 ± 9	15 ± 8	*<0.001

Epicardial Fat mm^2	1219 ± 653	532 ± 316	*0.002

* denotes p < 0.05

## Conclusions

Cardiac remodeling and dysfunction are present in children with obesity as evidenced by increased left ventricular mass and reduced longitudinal strain. These changes in structure and function in the heart are correlated with measures of systolic blood pressure and the amount of epicardial fat.

## Funding

This project was supported by a grant from the National Institute of General Medical Science (P20 GM103527) of the National Institutes of Health; the University of Kentucky Cardiovascular Research Center; the National Center for Research Resources and the National Center for Advancing Translational Sciences, National Institutes of Health (UL1TR000117), and contributions made by local businesses and individuals through a partnership between Kentucky Children's Hospital and Children's Miracle network. The content is solely the responsibility of the authors and does not necessarily represent the official views of the funding sources.

